# Spatial frequency discrimination learning in normal and developmentally impaired human vision

**DOI:** 10.1016/j.visres.2010.09.004

**Published:** 2010-11-23

**Authors:** Andrew T. Astle, Ben S. Webb, Paul V. McGraw

**Affiliations:** Visual Neuroscience Group, School of Psychology, University of Nottingham, Nottingham NG7 2RD, UK

**Keywords:** Amblyopia, Perceptual learning, Spatial frequency, Discrimination, Detection

## Abstract

Perceptual learning effects demonstrate that the adult visual system retains neural plasticity. If perceptual learning holds any value as a treatment tool for amblyopia, trained improvements in performance must generalise. Here we investigate whether spatial frequency discrimination learning generalises within task to other spatial frequencies, and across task to contrast sensitivity. Before and after training, we measured contrast sensitivity and spatial frequency discrimination (at a range of reference frequencies 1, 2, 4, 8, 16 c/deg). During training, normal and amblyopic observers were divided into three groups. Each group trained on a spatial frequency discrimination task at one reference frequency (2, 4, or 8 c/deg). Normal and amblyopic observers who trained at lower frequencies showed a greater rate of within task learning (at their reference frequency) compared to those trained at higher frequencies. Compared to normals, amblyopic observers showed greater within task learning, at the trained reference frequency. Normal and amblyopic observers showed asymmetrical transfer of learning from high to low spatial frequencies. Both normal and amblyopic subjects showed transfer to contrast sensitivity. The direction of transfer for contrast sensitivity measurements was from the trained spatial frequency to higher frequencies, with the bandwidth and magnitude of transfer greater in the amblyopic observers compared to normals. The findings provide further support for the therapeutic efficacy of this approach and establish general principles that may help develop more effective protocols for the treatment of developmental visual deficits.

## Research highlights

► Amblyopic observers show greater learning compared to normal observers. ► Learning transfers asymmetrically from high to low spatial frequencies. ► Spatial frequency discrimination learning transfers to contrast sensitivity. ► Principles established may help develop more effective treatment protocols.

## 1 Introduction

Critical, or sensitive, periods of visual development are post-natal windows of experience-dependent neural plasticity (Wiesel & Hubel, 1963). The anatomical and functional development of the visual system is characterised by a series of critical periods that have different start and end points in the developmental sequence (Daw, 1998; Harwerth, Smith, Duncan, Crawford, & von Noorden, 1986; LeVay, Wiesel, & Hubel, 1980). Disruption of visual input to one eye during the critical period(s) of visual development leads to dramatic structural changes in visual cortex and marked functional impairments of vision, known as amblyopia – commonly referred to as ‘lazy eye’ (see Ciuffreda, Levi, & Selenow, 1991).

Amblyopia is a common visual problem, affecting approximately 2–4% of the population (von Noorden, 1996). It is diagnosed by reduced vision in one, or occasionally both eyes, despite full optical correction and no evident ocular pathology (Ciuffreda et al., 1991; McKee, Levi, & Movshon, 2003). It remains the most common form of monocular vision loss in children (Attebo, Mitchell, & Smith, 1996; Simons, 1996), and accounts for the majority of children’s eye appointments in the UK (Stewart, Fielder, Stephens, & Moseley, 2002).

The neural site of the amblyopic deficit is widely thought to be primary visual cortex (Barnes, Hess, Dumoulin, Achtman, & Pike, 2001; Hubel & Wiesel, 1965; Kiorpes & McKee, 1999; Wiesel & Hubel, 1963). Until relatively recently, adult visual cortex had not been considered capable of retaining any of the experience-dependent neural plasticity so prominent during early visual development. However, it is now abundantly clear that experience can reshape visual brain function throughout the lifespan, and plasticity can be expressed in many different forms – from molecular and synaptic changes (e.g. Sengpiel, 2007) to complete reorganisation of topographic cortical maps (e.g. Buonomano & Merzenich, 1998; Merzenich et al., 1984).

A much studied behavioural manifestation of neural plasticity in normal vision is ‘perceptual learning’, where repeatedly practicing a challenging visual task can lead to substantial improvements in task performance over time. These effects have been widely documented in adulthood, well beyond the critical period(s) of development (Dosher & Lu, 1998; Fiorentini & Berardi, 1997; Folta, 2003; Schoups, Vogels, Qian, & Orban, 2001). One of the hallmarks of perceptual learning in normal vision is that improvements are strongly coupled to local visual attributes such as orientation, spatial frequency, retinal position, size and binocular disparity of a visual stimulus (Fiorentini & Berardi, 1980; O’Toole & Kersten, 1992; Schoups et al., 2001; Shiu & Pashler, 1992). The degree of specificity, or conversely, the degree of transfer of learning to untrained tasks, is thought to be dependent on the particular training procedures (Xiao et al., 2008, Zhang, Xiao, Klein, Levi, & Yu, 2010) and task difficulty, with greater specificity often found for more challenging judgements (e.g. Ahissar & Hochstein, 1997, 2004; Liu & Weinshall, 2000; Rubin, Nakayama, & Shapley, 1997). Others have found that the degree of transfer is related to precision demands of the transfer task (Jeter, Dosher, Petrov, & Lu, 2009), or commonality between the judgements (Webb, Roach, & McGraw, 2007), rather than the task difficulty during training. Whether this form of stimulus-coupled learning reflects experience-dependent neural plasticity at the level at which visual information is represented, “read-out”, or both, is still a matter of considerable debate (Dosher & Lu, 1998; Fahle, 2004; Fiorentini & Berardi, 1997; Folta, 2003; Gilbert, 1994; Law & Gold, 2008; Mollon & Danilova, 1996; Petrov, Dosher, & Lu, 2005; Saarinen & Levi, 1995; Schwartz, Maquet, & Frith, 2002; Shiu & Pashler, 1992; Xiao et al., 2008). What is clear though is that the mature visual brain is much more malleable than previously presumed.

Recent studies have shown significant perceptual learning effects in adults with amblyopia (for a review, please see Levi & Li, 2009). Marked improvements have been demonstrated in relative localisation (Levi & Polat, 1996; Sireteanu, Lagreze, & Constantinescu, 1993), contrast detection (Fronius, Cirina, Kuhli, Cordey, & Ohrloff, 2006; Polat, Ma-Naim, Belkin, & Sagi, 2004; Zhou et al., 2006), letter recognition (Chung, Li, & Levi, 2006; Levi, 2005), and grating resolution (Fronius et al., 2006). In contrast to the task-specific learning effects found in subjects with normal vision some studies have found that trained improvements in amblyopic visual performance generalise to untrained tasks and novel stimuli (Chung et al., 2006; Levi, Polat, & Hu, 1997; Polat, 2009; Polat, Ma-Naim, & Spierer, 2009; Polat et al., 2004). In one recent study, the bandwidth of learning (transfer) on a contrast detection task, resulting from training at a single spatial frequency was found to be much broader in observers with anisometropic amblyopia compared to normal observers (Huang, Zhou, & Lu, 2008). This suggests that the amblyopic visual system may be uncharacteristic with regard to the specific improvements often found in the normal visual system as a result of perceptual learning. The issue of generalisation, or lack of it, is of central importance in determining whether perceptual learning protocols are likely to offer a viable alternative or supplement to occlusion therapy.

Here we examine directly the generalisation of perceptual learning in adults with normal vision and adults with developmentally impaired vision (amblyopia). We ask if training on a spatial frequency discrimination task results in sensory improvements that differ between these two subject groups. We then measure the within-task generalisation to other spatial scales for both groups. Finally, we investigate whether improvements in spatial frequency discrimination transfer to contrast detection judgments across a similar range of spatial frequencies.

## 2 Methods

### 2.1 Observers

Eighteen adults with normal vision (19–28 years; 4 males, 14 females) and 17 with naturally occurring amblyopia (17–57 years; 9 males, 8 females) completed the study. All observers were naïve to the specific purposes of the experiment, provided written informed consent and were free to withdraw from the study at any time. They wore their full optical correction, which was determined prior to training. The experimental procedure was approved by a local ethics committee at the School of Psychology, The University of Nottingham. Table 1 provides clinical data for all of the amblyopic observers.

### 2.2 Stimuli

Stimuli were generated on a PC using custom software written in Python (Peirce, 2007) and displayed on a gamma-corrected IIyama Vision Master Pro 514 monitor with a resolution of 1024 × 768 and update rate of 100 Hz. A digital-to-analogue converter (Bits++, Cambridge Research Systems, Cambridge UK) was used to increase the dynamic range of luminance levels from 256 (8-bit) grey levels to 16,384 (14-bit) grey levels. The non-linear luminance response of the CRT display was corrected using an inverse gamma function, measured with a Minolta CS-110 photometer (Konica Minolta, Canada). The screen was viewed via a mirror at a distance of 5 m. The stimulus was a Gabor patch consisting of a vertical sinusoidal carrier modulated on a uniform background (90 cdm^−2^) and windowed by a two-dimensional Gaussian function. The mathematical expression describing a Gabor is

(1) $$L(x\text{,}y)=L_{m}\left\{1+C_{p}\cos[2\pi x\;f_{c}+\theta_{c}]\times \exp\left[-\frac{1}{2}\left(\frac{x}{\sigma_{x}}\right)-\frac{1}{2}{\left(\frac{y}{\sigma_{y}}\right)}^{2}\right]\right\}\text{,}$$

where *L*_*m*_ is the mean luminance of the display, *C_p_* is the peak contrast of the Gabor, *f_c_* is the carrier spatial frequency, and *σ_x_* and *σ_y_* are standard deviations of the Gaussian envelope. For the contrast sensitivity task the standard deviation of the Gaussian was fixed at 3.08 deg and the contrast level varied. For spatial frequency discrimination measurement, the standard deviation of the Gabor was randomly jittered in the range of 2.93–3.25 deg with Michelson contrast fixed at 90%. The phase of the carrier (*θ*) was also randomly jittered (0–180 deg). Stimuli were presented for 200 ms and separated by a 500 ms interval containing a blank screen of mean luminance (90 cdm^−2^).

### 2.3 Procedure

Observers viewed the screen with their non-dominant or amblyopic eye while their head was secured in a fixed position using a forehead and chin rest. The contralateral eye was occluded and testing was carried out in a darkened room. Auditory feedback was given for incorrect responses.

The experiment consisted of three phases: pre-training, training and post-training. Pre-training and post-training phases were identical for all observers and involved measurement of spatial frequency discrimination and contrast sensitivity. Amblyopic and normal observers were randomly assigned to one of three groups. For the training phase, each group was trained on a spatial frequency discrimination task at a single reference frequency of 2, 4 or 8 c/deg for a period of 10 days.

Spatial frequency discrimination was measured using a 2 alternate forced choice task (2AFC) procedure. Each trial consisted of two 200 ms intervals separated by 500 ms. One interval contained the *reference* spatial frequency, the other the *comparison* spatial frequency. The comparison spatial frequency was varied initially using a descending 1-down, 1-up staircase and an ascending 1-up, 1-down staircase in turn. When an incorrect response was recorded, staircases changed to a descending 3-down, 1-up staircase and an ascending 3-up, 1-down staircase respectively. These staircases terminated after seven reversals and therefore the number of trials per run varied (but was approximately 50 trials). Just-noticeable differences (JND’s) were calculated by taking the geometric mean of the spatial frequency difference between the reference and comparison frequencies for the last four reversals of each staircase. For the pre- and post-training phases spatial frequency discrimination was measured at a range of reference frequencies (1, 2, 4, 8, and 16 c/deg). A block of trials corresponding to one reference spatial frequency was completed before moving onto the next spatial frequency. The order in which the blocks were completed was randomised for all subjects.

Contrast sensitivity was sampled at 6 spatial frequencies (0.5, 1, 2, 4, 8 and 16 c/deg) using a 2AFC procedure. Each trial consisted of two 200 ms intervals separated by 500 ms. One interval contained a Gabor patch; the other contained a mean luminance background. Observers indicated the interval containing the Gabor patch via a keyboard response. The contrast of the test patch was modulated according to a 3-down, 1-up staircase, which terminated after seven reversals. Contrast sensitivity was taken as the reciprocal of the geometric mean of the contrast threshold for the last four reversals. A block of trials corresponding to one reference spatial frequency was completed before moving onto the next spatial frequency. The order in which the blocks were completed was randomised for all subjects.

### 2.4 Data analysis

**Fig. 1:**
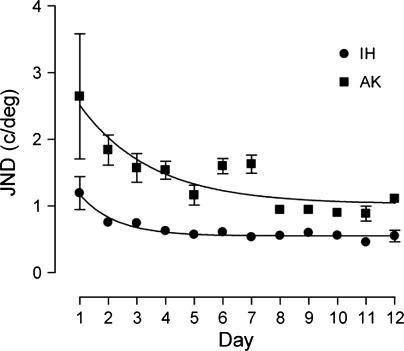
Example learning curves for two observers that trained on a spatial frequency discrimination task at a reference frequency of 8 c/deg. Observer IH (circles) has normal vision, observer AK (squares) is amblyopic. Error bars represent the standard error of the mean (SEM). Smooth curves through the data points are the best fitting solutions of Eq. (2).

**Fig. 2:**
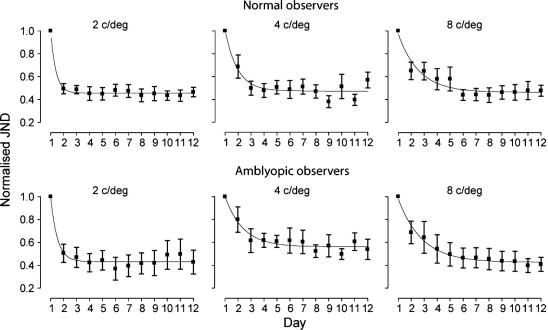
Mean normalised learning curves for the different spatial frequency training groups. Mean performance for each group has been normalised such that the mean JND on day 1 (pre-training) was set to unity. Error bars represent SEM. Smooth curves through the data points are the best fitting solutions of Eq. (2).

Learning curves (Figs. 1 and 2) were fitted with a one-phase exponential decay function of the form:

(2) $$\text{JND}=s\times 10^{-\mathrm{kf}}+p\text{,}$$

where JND is the just noticeable difference, *s* is the span, *f* is spatial frequency, *p* is the plateau and *k* is the rate constant.

Contrast sensitivity data (see Fig. 7) were fitted with a double exponential function of the form:

(3) $$S=a\;f^{b}\;e^{-\mathrm{cf}}\text{,}$$

where *S* is contrast sensitivity, *f* is spatial frequency and *a*, *b* and *c* are fitted parameters (Kiorpes & McKee, 1999).

Pre/post-training JND data, plotted as a function of trained spatial frequency (see Fig. 6) for both subject groups, was fitted with the descriptive function:

(4) $$R=M+R_{\max}\exp\left(\frac{-{\left(x-x_{\max}\right)}^{2}}{\sigma (x)}\right)\text{,}$$

where the parameter *σ*(*x*) can be either *σ*− or *σ*+, depending on whether *x* < *x*_max_ or *x* > *x*_max_, respectively. The parameter *M* can be either *M*− or *M*+ depending on whether *x* < *x*_max_ or *x* > *x*_max_ respectively. *M*+, *M*−, *R*_max_, *x*_max_, *σ*− and *σ*+ are free parameters (Freeman, Durand, Kiper, & Carandini, 2002; Webb, Dhruv, Solomon, Tailby, & Lennie, 2005). The fit of this function to the amblyopic data was scaled and fitted to the normal data. We obtained the fits by minimising the mean square error between the model and the data using the fmincon function of the Optimisation Toolbox for Matlab (version 6.5; Mathworks, Natick, MA).

## 3 Results

### 3.1 Spatial frequency discrimination learning in normal and amblyopic observers

Repeatedly practicing the spatial frequency discrimination task improved performance at the trained spatial frequency for both normal and amblyopic observers. Fig. 1 shows two example learning curves; one for an observer with normal vision (IH) and one for an observer with amblyopia (AK). Both observers trained at a reference spatial frequency of 8 c/deg. The amblyopic observer had a higher initial JND (2.64 c/deg) prior to training compared to the normal participant (1.19 c/deg). The size of the overall improvement in discrimination performance was also greater for the amblyopic observer (1.54 c/deg) compared to the normal (0.64 c/deg). However, the rate of learning across the training sessions was approximately equivalent for the two observers (*k* = 0.58 and 0.54 for AK and IH respectively).

The mean age of the amblyopic observers in each group was 38, 42 and 32 years for the 2, 4, and 8 c/deg reference-frequency groups, while for the visually normal observers it was 21, 23 and 20 years for the 2, 4, and 8 c/deg reference-frequency groups respectively. The mean visual acuity of amblyopic observers was 0.50, 0.52 and 0.42 logMAR for the 2, 4, and 8 c/deg reference-frequency groups while the mean visual acuity for the normal observers was −0.08, −0.04 and −0.02 logMAR for the 2, 4, and 8 c/deg reference-frequency groups respectively. Fig. 2 shows mean learning curves normalised to performance on the first day of training for normal and amblyopic observers at the three reference training frequencies. All groups showed improvements in performance at the spatial frequency they trained at. The mean magnitude of learning, which is commonly expressed as the ratio of post-learning threshold to pre-learning threshold (PPR), was 0.5 for observers with normal vision (*t*(17) = 7.81, *p* < .001) and 0.38 for observers with amblyopia (*t*(16) = 5.40, *p* < .001). The difference between the improvement (PPR) in normal and amblyopic observers was significant (*t*(34) = 3.44, *p* < .01).

**Fig. 3:**
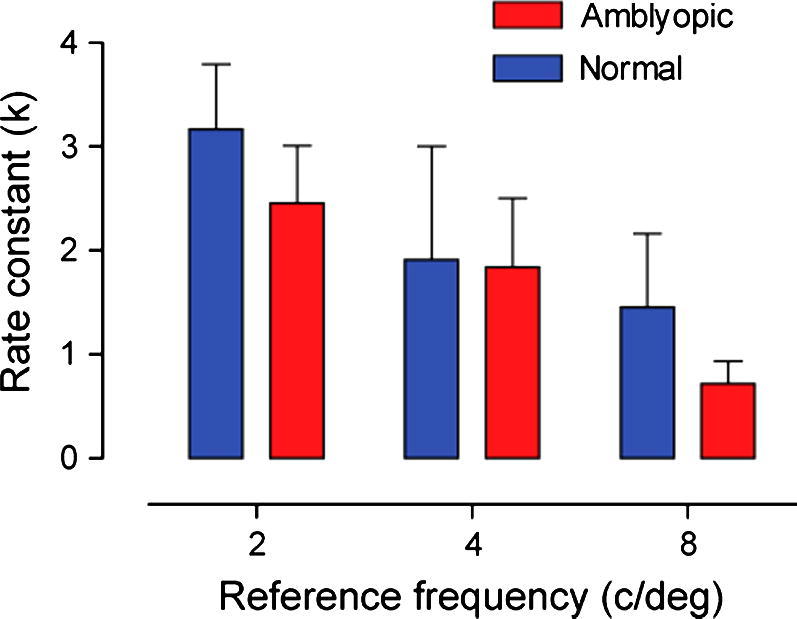
The rate of learning for each subject group, at each of the three reference training spatial frequencies (2, 4 and 8 c/deg). A higher *k*-value represents a more rapid rate of learning. Error bars represent SEM.

The rate of learning, quantified by the rate constant (*k*) from the exponential function described in Eq. (2), showed that learning was faster (higher *k*-value) for lower reference spatial frequencies. This can be seen in Fig. 3^1^ where the rate constants are presented for each subject group at the three reference training frequencies. The rate of learning was similar between normal and amblyopic observers who trained at 2 or 4 c/deg. However, amblyopic observers who trained at 8 c/deg showed a slower rate of improvement compared with their visually normal counterparts.

**Fig. 4:**
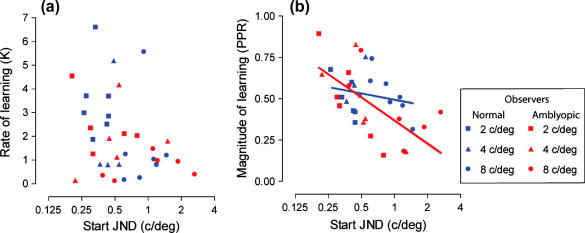
Scatterplots showing (a) rate of learning and (b) magnitude of learning versus start JND for all normal (blue symbols) and amblyopic observers (red symbols). There is no obvious relationship between the rate of learning and each observer’s individual threshold at the start of training. In contrast, the magnitude of learning is proportional to starting threshold, particularly for the amblyopic observers. Data in (b) were fitted with the following equation: *y* = *m* × ln (*x*) + *c* where *y* is the magnitude of learning, *m* is the slope of the curve, *x* is the start JND and *c* is a constant. The slope of the amblyopic observer curve differs significantly from zero (slope = −0.20; 95% CI, −0.33 to −0.07) but that of the normal observer curve does not (slope = −0.06; 95% CI, −0.18 to 0.07).

One possible explanation for the slower rate of learning found for the amblyopic observers at the highest reference training frequency (8 c/deg) may be that their pre-learning discrimination threshold is poorer at this particular frequency. To explore this further we plotted the rate of learning against the JND obtained at the start of training. These data are presented in Fig. 4a and show that for both groups the two factors are unrelated. For example, observers with a starting JND of 0.5 c/deg have rate constants that can vary by a factor of about 10. Similarly, subjects with similar rates of learning had starting discrimination thresholds that varied by a factor of 4 (e.g. *k* = 1).

Unlike the rate of learning, we found a strong relationship between the amount of learning and starting JND. This relationship is shown in Fig. 4b, where the trained improvement in discrimination performance is expressed in terms of pre–post JND ratio (JND post-training divided by JND pre-training (PPR)) and plotted against each individual’s starting JND. When improvements in threshold are expressed in this way a PPR of 1 represents no change in performance with training (i.e. no learning) and a PPR of 0.5 represents a 50% improvement in discrimination threshold. The amblyopic observers show a strong relationship (slope = −0.20; *r*^2^ = 0.42) between starting JND and the amount of learning. A much weaker (slope = −0.06; *r*^2^ = 0.05) trend was observed for the visually normal individuals. To ensure that this finding for the amblyopic observers does not simply result from the systematic variation in thresholds at different reference frequencies, we confirmed that the proportional relationship between starting JND and the magnitude of learning was present for each individual reference frequency group (2 c/deg: slope = −0.48, *r*^2^ = 0.82; 4 c/deg: slope = −0.30, *r*^2^ = 0.54; 8 c/deg: −0.19, *r*^2^ = 0.45). Therefore, amblyopic observers learned more when their start JND was higher. In contrast, the relationship between the magnitude of learning and visual acuity prior to training was very weak for both normal (slope = −0.26, *r*^2^ = 0.04) and amblyopic (slope = −0.41, *r*^2^ = 0.08) observers.

### 3.2 Transfer of spatial frequency discrimination learning

**Fig. 5:**
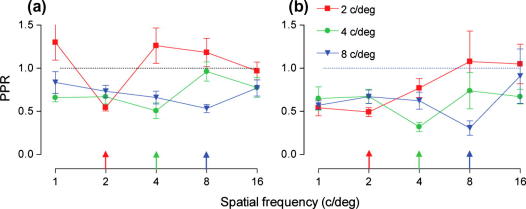
Pre–post ratio plotted against spatial frequency for (a) normal observers and (b) amblyopic observers. Points lying below the dotted line (PPR = 1) denote an improvement in performance. Error bars represent SEM.

To determine how specific the improvements in spatial frequency discrimination learning are to the trained spatial frequency, we plotted the pre–post JND ratio (PPR) against spatial frequency. Fig. 5 shows that for both normal and amblyopic observers the greatest improvement in performance on the spatial frequency discrimination task was found at the trained spatial frequency. For normal observers, the degree of improvement was considerable (∼50%) and broadly similar across all reference frequencies (indicated by the coloured arrows in Fig. 5). Amblyopic observers who trained with higher reference spatial frequencies (4 or 8 c/deg) showed systematically greater learning than those trained with the lowest reference spatial frequency (2 c/deg). Comparing the magnitude of learning across groups, at the lowest reference frequency the magnitude of learning was similar (∼50%), but amblyopic observers showed greater learning for the two higher reference frequencies (4 and 8 c/deg). The data from the amblyopic observers, and to a lesser extent the normal observers, show that the transfer of learning to untrained spatial frequencies is asymmetric, with performance improvements maintained for lower, but not higher spatial frequencies.

**Fig. 6:**
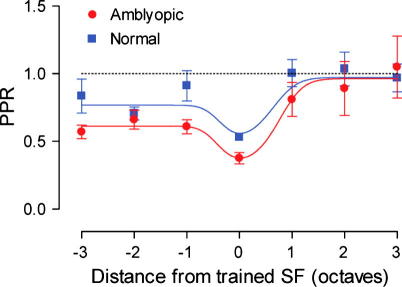
PPR data for normal (blue squares) and amblyopic (red circles) observers collapsed across trained spatial frequency. Points lying below the dotted line (PPR = 1) denote an improvement in performance. Error bars represent SEM. Both groups show more transfer of learning to frequencies lower than the trained spatial frequency. Smooth curves through the data points are the best fitting solutions of Eq. (4).

To quantify the asymmetry in the transfer of learning the data from Fig. 5 has been re-plotted in Fig. 6, with the *x*-axis transformed such that all of the training functions are collapsed on one another. This has the effect of realigning the coloured arrows in Fig. 5 to a single point in Fig. 6. Now, 0 on the *x*-axis represents the improvements at the trained spatial frequency, regardless of reference frequency trained at. All other data points are then represented in terms of their distance (in octaves) from the trained spatial frequency.

The data in Fig. 6 have been fitted with the Gaussian function described in Eq. (4). This figure reveals an important new principle for spatial frequency discrimination learning: the transfer of learning is unidirectional, cascading from higher to lower spatial scales but not the reverse. This is true for both the normal and amblyopic observers. The curve fitted to the normal data is simply a scaled version of that fitted to the amblyopic data set. The scaling factor was 0.48, and was derived by a minimisation procedure (see Section 2). Therefore, for normal and amblyopic observers there exists a simple quantitative difference in the amount of learning at the trained spatial frequency and this difference is maintained for all test frequencies lower than the training frequency.

### 3.3 Transfer to contrast sensitivity

**Fig. 7:**
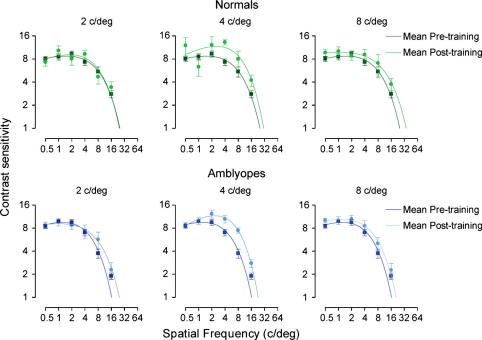
Mean contrast sensitivity functions for each of the training groups before and after spatial frequency discrimination training. Error bars represent SEM. Smooth curves through the data points are the best fitting solutions of Eq. (3).

To assess whether learning on a spatial frequency discrimination task transfers to a contrast detection task we measured contrast sensitivity in all observers before and after training. Fig. 7 shows pre- and post-training contrast sensitivity data for the six different training groups. Little change in peak contrast sensitivity was found for amblyopic and normal observers trained at reference frequencies of 2 and 8 c/deg. However, both amblyopic and normal observers who trained at a reference frequency of 4 c/deg showed a contrast sensitivity improvement, driven primarily by changes at or around the trained spatial frequency.

To quantify changes in detection performance in more detail the area under the contrast sensitivity curve was calculated for each observer before and after training. An increase in the area indicated an improvement in contrast sensitivity. There was a statistically significant increase in the area under the contrast sensitivity function for amblyopic observers (pre/post-training area under curve = 0.76, *t*(16) = 3.40, *p* < .01). There was also a statistically significant improvement for observers with normal vision (pre/post-training area under curve = 0.67, *t*(17) = 2.32, *p* < .05).

**Fig. 8:**
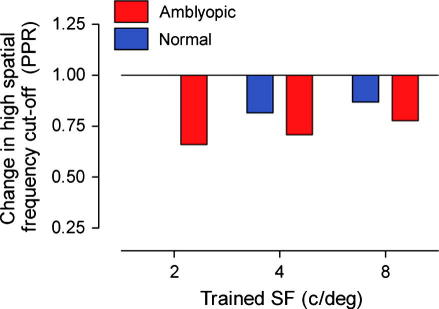
Change in contrast sensitivity expressed as a ratio (PPR) of high-frequency cut-off values for each of the training groups before and after spatial frequency discrimination training. The normal group that trained at 2 c/deg showed no transfer of learning between tasks. However, all other training groups showed improvements in this measure.

The curve fit to each data set in Fig. 7 was extrapolated to reveal the high spatial frequency cut-off before and after training. This difference is often used as an index for improvement in contrast sensitivity and is plotted for each of the training groups in Fig. 8. The plot shows that virtually all groups show a transfer in learning for this measure. The only exception is the group of normal subjects that trained at 2 c/deg, where no change in high-frequency cut-off was found after training on the spatial frequency discrimination task.

**Fig. 9:**
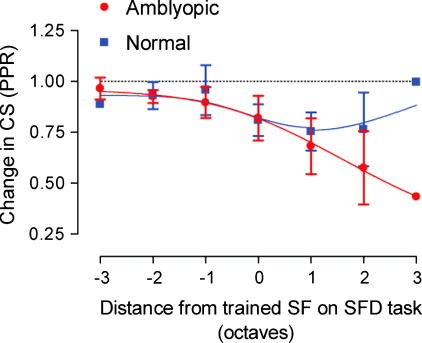
Transfer of learning from spatial frequency discrimination to contrast sensitivity (CS). Pre–post ratio (PPR) is plotted as a relative distance (in octaves) from the spatial frequency (SF) at which observers were trained on the spatial frequency discrimination (SFD) task. PPR values were calculated from the fit to the mean group data shown in Fig. 7 (ratio of CS prior to SFD training to CS after training). Error bars represent SEM. No error bars are shown at 3 octaves, since only one PPR value contributes to this point for both the amblyopic and normal observers (CS at 16 c/deg for groups trained at 2 c/deg). Smooth curves through the data points are the best fitting solutions of Eq. (4).

To reveal the asymmetry of learning in the spatial frequency discrimination task we plotted the change in performance at each spatial frequency resulting from training at a single reference frequency. In Fig. 9, we present a similar analysis, except that the pre–post ratio (PPR) for contrast sensitivity (CS) is plotted as a relative distance (in octaves) from the spatial frequency at which observers were trained on the spatial frequency discrimination task (SFD). PPR values were derived from the curve fit to the mean group data shown in Fig. 7 (ratio of CS prior to SFD training to CS after training). From this figure it is clear that most of the transfer of learning, in terms of improvement in detection threshold, occurs for spatial frequencies higher than the spatial frequency at which discrimination training took place. Moreover, the bandwidth of this transfer between tasks appears to be much broader in the amblyopic than in the normal observers. Unlike spatial frequency discrimination learning, the generalisation of learned improvements to detection performance appears to move in the opposite direction, from lower to higher spatial frequencies.

## 4 Discussion

We found significant improvements in adult performance on a spatial frequency discrimination task as a result of repeated practice. This is consistent with many other studies that have documented improvements on a range of visual tasks, and who have attributed such improvements to the retention of cortical plasticity well into adulthood (Dosher & Lu, 1999; Fiorentini & Berardi, 1980; Gilbert, 1994; Karni & Sagi, 1991; Poggio, Fahle, & Edelman, 1992; Saarinen & Levi, 1995). Indeed, the magnitude of improvement we found in our normal subjects (trained at 4 c/deg) was in excellent agreement to that reported previously for a group trained at a similar spatial frequency (4.25 c/deg) (Meinhardt, 2001). We set out to compare improvements on this task between adults with normal vision and those with amblyopia. Recent studies have highlighted important differences in learning-based improvements between these groups, including the magnitude of learning (Chen, Chen, Fu, Chien, & Lu, 2008; Polat et al., 2004), rate of learning (Li, Klein, & Levi, 2008) and bandwidth of learning (Huang et al., 2008). These differences raise the question as to whether the mechanism facilitating improvements associated with perceptual learning in each population are themselves different or whether they might reflect variation in the underlying structure and function of the normal and amblyopic visual systems.

The rate of learning was faster for observers who trained at lower reference frequencies and reduced as the reference spatial frequency was increased. This was particularly evident in the amblyopic group. Previous research has shown that the rate of learning is directly related to the degree of amblyopia – quantified as a difference in positional acuity measures between eyes. Specifically, subjects with deeper amblyopia (i.e. poorer starting acuity in the amblyopic eye) take much longer to reach asymptotic performance (Li et al., 2008). We investigated whether the starting JND on a spatial frequency discrimination task was diagnostic of the rate of learning, but found no obvious relationship (Fig. 4a). This was also true when we considered other measures of initial visual performance such as logMAR visual acuity. It should be noted that in the Li et al., study several subjects took considerable periods of time to reach asymptotic performance – up to 50 h in some instances – which is well beyond the training periods we used (10 training sessions lasting approximately 5 h). Moreover, in some subjects, performance improvements were characterised by a series of exponential decay functions that occur in sequence as training proceeds. In such cases, our measure of rate constant (*k*) over a relatively short training period is unlikely to capture these additional improvements that accrue over extended training periods (Li, Provost, & Levi, 2007; Li et al., 2008).

The magnitude of spatial frequency discrimination learning, on the other hand, was greater for individuals with poorer starting performance and this relationship was stronger in amblyopic compared to normal observers. Previous reports of perceptual learning effects in visually normal subjects (Fahle & Henke-Fahle, 1996) have observed this relationship. Our results are also consistent with other studies that have found greater amounts of learning in more severe cases of amblyopia (compared to those with mild amblyopic deficits) using different visual tasks (Chen et al., 2008; Li et al., 2008; Polat, 2008; Polat et al., 2004). We found that for spatial frequency discrimination learning, the starting threshold on the task is a reasonable indicator of the likely improvements that can be expected to result from a fixed period of training, but not of the time required to realise these improvements.

We were able to determine the direction of transfer of improvements to neighbouring (non-trained) spatial frequencies on the task and found an asymmetry in favour of lower spatial frequencies. Previously, Meinhardt (2001), trained normal observers at a reference frequency of 4.25 c/deg and found no evidence of transfer to a single test frequency of 2.25 c/deg. This may be explained by the fact that in normal observers, the transfer of learned improvements to lower spatial frequencies (e.g. −1 octave in Fig. 6) is fairly modest, but is evident when compared against transfer in the opposite direction (e.g. +1 octave in Fig. 6). Our data show that the transfer of learning translates from higher spatial frequencies to lower and the bandwidth of this transfer is approximately equivalent for each reference spatial frequency. This asymmetry is clearly more evident in the amblyopic group. However, the fact that the data from both groups are well described by a single function that differs only by a scaling factor strongly suggests a common mechanism drives this pattern of results in both subject groups. We found no differences in the pattern of asymmetrical transfer of learned improvements between the different amblyopic sub-groups (anisometropic, strabismic and mixed amblyopia).

It is widely accepted that the ability to discriminate between different spatial frequencies is determined by analysing the relative outputs of spatial frequency channels (Campbell, Nachmias, & Jukes, 1970a). For discrimination judgments around any particular spatial frequency, the neurons that carry the most accurate information are not centred on the reference spatial frequency of interest, but are found tuned away from the discrimination boundary. Therefore, the contribution of any spatial frequency selective neurons to discrimination judgments is not maximal at the point where it is most sensitive, but occurs where the sensitivity changes most rapidly (Regan & Beverley, 1983). In support of this notion, patients with multiple sclerosis, where sensitivity deficits are spatial frequency selective, show normal discrimination performance at spatial scales where sensitivity is depressed and abnormal discrimination is found in regions where sensitivity is ostensibly normal (Regan, Bartol, Murray, & Beverley, 1982). In the present study, we found that there was an improvement in contrast sensitivity at the trained spatial frequency, and at higher spatial frequencies, where little spatial frequency discrimination learning took place. In contrast, there was little change in contrast sensitivity performance at spatial frequencies lower than the trained frequency, where the majority of spatial frequency discrimination learning transfer was found. This strengthens the notion that detection and discrimination judgments can be dissociated in frequency space and are governed by the operation of different frequency channels (Campbell, Nachmias, & Jukes, 1970b).

Important differences exist between discrimination mechanisms for spatial frequency and the discrimination of orientation or motion direction. For the latter cases, the neurons carrying the most accurate information are tuned away but on both sides of the discrimination boundary (Hol & Treue, 2001; Regan & Beverley, 1985). However, for spatial frequency discrimination the most informative neurons are usually tuned to spatial frequencies one octave below the discrimination boundary. This has been confirmed by showing that adaptation to a particular spatial frequency results in peak deficits in discrimination performance that do not coincide with the adapted frequency, but instead are found at twice the adapting frequency (Regan & Beverley, 1983). To accommodate our findings within this framework, we would need to assume that when subjects train at a particular spatial frequency, operational changes are implemented in mechanisms that are sensitive to a spatial frequency one octave lower and that degree of change is related to initial levels of internal noise. The precise mechanism for this change is not yet understood. However, it need not be viewed in terms of changing the sensitivity profiles of any underlying channels but could simply reflect a reduction in internal neural noise or retuning of the weights of channel outputs to a read-out mechanism (e.g. Dosher & Lu, 1998; Gold, Bennett, & Sekuler, 1999; Li & Levi, 2004). Regan and Beverley (1983) showed that post-adaptation discrimination performance is most impaired for frequencies about an octave higher than the adapting frequency, though changes in discrimination threshold occur across a range of spatial frequencies (approximately 2 octaves). Our training induced improvements in spatial frequency discrimination could be described as an increase in the peak of this post-adaptation cost function and an associated broadening of its bandwidth. This sort of change, coupled with the fact that spatial frequency discrimination judgments already show an asymmetry in the locus of the most informative mechanisms in the frequency dimension (i.e. lower than discrimination frequency), would be qualitatively consistent with the largest improvements occurring at the trained frequency and the transfer of learning effects to mechanisms that operate at lower spatial scales.

Alternatively, the relatively large bandwidth of transfer (at least 3 octaves) could be due to an asymmetric spread of learning from the trained spatial frequency to lower spatial frequencies, combined with relatively rapid learning at lower spatial frequencies. We have shown that the rate of spatial frequency discrimination learning is greater for lower spatial frequencies, with relatively large degrees of learning being found within the first few sessions. If individuals also show a large amount of learning from the pre-training session to the post-training session (i.e. learning over two sessions), at these lower spatial frequencies, without receiving any specific training at these frequencies in the intervening sessions, this could lead to a greater apparent bandwidth of learning. However, the data presented in Fig. 6 show that the bandwidth of transfer is not critically dependant on training at any particular reference spatial frequency and therefore a more rapid rate of learning at the lowest spatial frequencies is unlikely to provide a sufficient account of the data.

The finding that spatial frequency discrimination learning transfers from high to low spatial frequencies is novel, and holds significant ramifications for use of perceptual learning as a potential clinical tool (Levi, 2005; Levi & Li, 2009; Polat, 2009; Polat et al., 2009; Webb, McGraw, & Levi, 2006). This pattern of transfer was found, not only in amblyopic observers but also in observers with normal vision. There is also evidence that this pattern may hold for other tasks. For example, learning on a contrast sensitivity task near the high spatial frequency cut-off point leads to a transfer of improvement to considerably lower spatial frequencies but does not lead to improvements in letter acuity (Huang et al., 2008). Additionally, training on a contrast-defined letter identification task, near the contrast threshold, does not lead to improvements in visual acuity (Chung et al., 2006). One way of reconciling these findings is to presume a unidirectional spread of learning from high to low spatial frequencies (Levi & Li, 2009a).

We found improvements in contrast sensitivity as a result of spatial frequency discrimination training. These improvements were evident at the trained spatial frequency and at higher spatial frequencies. Our data show that the bandwidth of transfer to contrast sensitivity is greater in the amblyopic subject group. Given that a deficit in contrast sensitivity at high spatial frequencies is characteristic of human amblyopia, it is perhaps unsurprising that improvements are found in this region. Perceptual training of contrast sensitivity judgments has been found to result in enhancements to the contrast sensitivity function (Huang et al., 2008). More specifically, amblyopic observers exhibited a greater bandwidth and magnitude of learning relative to normal subjects (Huang et al., 2008). Here, we replicate this important finding, but do so using a supra-threshold discrimination task. It would be interesting to know if this relationship is bi-directional: namely, would training on a detection task (e.g. contrast sensitivity task) at a fixed spatial scale produce benefits in spatial frequency discrimination?

These results establish an important canon for the design of learning-based therapies: most benefit will be derived from training observers at the highest spatial frequency they can detect, in the knowledge that improvements are likely to spread to lower spatial frequencies, as opposed to training at lower spatial frequencies, where we find little transfer to higher spatial frequencies. Furthermore, our results show that differences in performance between normal and amblyopic observers are merely quantitative and the mechanisms driving these improvements are likely to be common. Therefore, the normal visual system is a useful test bed for determining the general rules of learning, for future implementation in learning-based therapies for treating amblyopia in adults.

## Figures and Tables

**Table 1:** Clinical details of amblyopic observers (Strab = strabismus, NMD = no movement detected, RSOT/LSOT = right/left esotropia, RXOT/LXOT = right/left exotropia).

Observer	Base SF	Age (years)	Gender	Amblyopic eye	Refractive error	VA (logMAR)	Strab	Type of amblyopia	Treatment history
JB	2	21	M	L	R −0.50 DS	−0.10	NMD	Anisometropic	Spectacles
					L +1.00/−0.50 × 105	0.12			

IMB	2	41	M	L	R + 0.50 DS	−0.10	NMD	Anisometropic	Spectacles, occlusion
					L +5.50/−0.50 × 30	0.62			

LBM	2	28	F	L	R +4.25/−1.50 × 115	0.10	LXOT	Strabismic and anisometropic	Strabismus surgery, occlusion, spectacles
					L +5.00/−0.50 × 70	0.52			

NE	2	47	F	L	R pl	−0.08	NMD	Anisometropic	Occlusion
					L −3.50/−0.75 × 150	0.62			

PS	2	53	F	L	R pl	0.00	NMD	Anisometropic	Spectacles, occlusion
					L +2.50/−1.00 × 50	0.50			

HB	2	39	F	L	R +0.75 DS	−0.02	NMD	Anisometropic	Spectacles, occlusion
					L +2.75 DS	0.38			

JAC	4	45	M	L	R +0.50/−0.50 × 180	0.02	LSOT	Strabismic	Strabismus surgery, occlusion, atropine
					L pl/−0.50 × 60	0.62			

BL	4	34	M	L	R −0.50 DS	0.00	LSOT	Strabismic	Spectacles, occlusion
					L −0.50 DS	0.30			

DG	4	30	M	R	R +3.50/−2.00 × 120	0.50	NMD	Anisometropic	None
					L +1.25/−0.75 × 110	0.00			

RPS	4	44	M	L	R −0.50 DS	0.02	LSOT	Strabismic	None
					L −0.50/−0.25 × 110	0.50			

SLR	4	57	F	R	R +4.50/−0.75 × 130	0.70	RSOT	Strabismic and anisometropic	Spectacles, occlusion
					L +2.50/−0.50 × 180	0.04			

AK	8	28	F	L	R −4.00/−0.75 × 10	0.04	LSOT	Strabismic and anisometropic	Spectacles, occlusion
					L −4.00/−1.75 × 175	0.30			

DP	8	17	M	R	R −13.00/−1.00 × 135	0.60	RSOT	Strabismic	Occlusion
					L −13.00/−2.00 × 10	0.30			

JA	8	46	F	R	R +1.00/−0.75 × 180	0.30	NMD	Anisometropic	Spectacles, occlusion
					L pl	−0.10			

JC	8	43	F	R	R +5.00/−0.50 × 80	0.32	NMD	Anisometropic	Spectacles
					L +3.50/−0.50 × 40	0.08			

MT	8	33	M	R	R +1.75/−1.50 × 180	0.58	RXOT	Strabismic and anisometropic	Spectacles, occlusion
					L +0.50/−0.50 × 100	0.00			

SCJ	8	22	M	R	R +3.00/−1.50 × 150	0.42	NMD	Anisometropic	None
					L −0.25 DS	−0.18			

## Footnotes

1 For interpretation of colour in Figs. 3–9, the reader is referred to the web version of this article.
